# Safety and incremental prognostic value of stress cardiovascular magnetic resonance in patients with known chronic kidney disease

**DOI:** 10.1186/s12968-023-00939-8

**Published:** 2023-06-12

**Authors:** Théo Pezel, Thierry Unterseeh, Thomas Hovasse, Francesca Sanguineti, Philippe Garot, Stéphane Champagne, Solenn Toupin, Tania Ah-Sing, Alyssa Faradji, Martin Nicol, Lounis Hamzi, Jean Guillaume Dillinger, Patrick Henry, Valérie Bousson, Jérôme Garot

**Affiliations:** 1 Université de Paris Cité, Service de Cardiologie, Hôpital Lariboisière–APHP, Inserm UMRS 942, 75010 Paris, France; 2 CMR Department-ICPS, Institut Cardiovasculaire Paris Sud, Cardiovascular Magnetic Resonance Laboratory, Hôpital Privé Jacques CARTIER, Ramsay Santé, 6 Avenue du Noyer Lambert, 91300 Massy, France; 3 Université de Paris Cité, Service de Radiologie, Hôpital Lariboisière–APHP, 75010 Paris, France; 4 Siemens Healthcare France, 93200 Saint-Denis, France

**Keywords:** Cardiovascular magnetic resonance, Stress testing, Chronic kidney disease, Unrecognized myocardial infarction, Myocardial ischemia, Cardiovascular events, Prognosis

## Abstract

**Background:**

Cardiovascular disease (CVD) is the main cause of mortality in patients with chronic kidney disease (CKD). Although several studies have demonstrated the consistently high prognostic value of stress cardiovascular magnetic resonance (CMR), its prognostic value in patients with CKD is not well established. We aimed to assess the safety and the incremental prognostic value of vasodilator stress perfusion CMR in consecutive symptomatic patients with known CKD.

**Methods:**

Between 2008 and 2021, we conducted a retrospective dual center study with all consecutive symptomatic patients with known stage 3 CKD, defined by estimated glomerular filtration rate (eGFR) between 30 and 60 ml/min/1.73 m^2^, referred for vasodilator stress CMR. All patients with eGFR < 30 ml/min/1.73 m^2^ (n = 62) were excluded due the risk of nephrogenic systemic fibrosis. All patients were followed for the occurrence of major adverse cardiovascular events (MACE) defined as cardiac death or recurrent nonfatal myocardial infarction (MI). Cox regression analysis was used to determine the prognostic value of stress CMR parameters.

**Results:**

Of 825 patients with known CKD (71.4 ± 8.8 years, 70% men), 769 (93%) completed the CMR protocol. Follow-up was available in 702 (91%) (median follow-up 6.4 (4.0–8.2) years). Stress CMR was well tolerated without occurrence of death or severe adverse event related to the injection of gadolinium or cases of nephrogenic systemic fibrosis. The presence of inducible ischemia was associated with the occurrence of MACE (hazard ratio [HR] 12.50; 95% confidence interval [CI] 7.50–20.8; p < 0.001). In multivariable analysis, ischemia and late gadolinium enhancement were independent predictors of MACE (HR 15.5; 95% CI 7.72 to 30.9; and HR 4.67 [95% CI 2.83–7.68]; respectively, both p < 0.001). After adjustment, stress CMR findings showed the best improvement in model discrimination and reclassification above traditional risk factors (C-statistic improvement: 0.13; NRI = 0.477; IDI = 0.049).

**Conclusions:**

In patients with known stage 3 CKD, stress CMR is safe and its findings have an incremental prognostic value to predict MACE over traditional risk factors.

**Supplementary Information:**

The online version contains supplementary material available at 10.1186/s12968-023-00939-8.

## Introduction

Cardiovascular disease (CVD) is the main cause of mortality in patients with chronic kidney disease (CKD) with a risk of cardiac death more than ten times higher than the general population [1]. CKD and coronary artery disease (CAD) share common risk factors and previous studies reported a prevalence of obstructive CAD of more than 50% in patients with CKD. As estimated glomerular filtration rate (eGFR) declines below ∼ 60 ml/min/1.73 m^2^, the probability of developing CAD increases linearly [2–4]. Beyond the role of traditional CVD risk factors, such as diabetes and hypertension, patients with CKD are also exposed to other non-traditional risk factors related to uremia, including oxidative stress, inflammation, and abnormal calcium-phosphorus metabolism [4]. Therefore, it could be relevant to detect CAD in patients with eGFR < 60 ml/min/1.73 m^2^, who might benefit from additional interventions. However, several reports indicate that the clinical presentation of CAD is often atypical. Indeed, an “oligo-symptomatic” presentation is frequent with only 44% of patients with CKD who present with acute myocardial infarction (MI) reporting typical chest pain compared with 72% of patients with normal kidney function [5]. Several reports showed that stress testing has reduced accuracy for detecting CAD in CKD, with a higher rate of both false-negative and false-positive tests [4]. Despite the relative accuracy of noninvasive stress testing in CKD, these methods appear interesting for risk stratification. The risk of death is nearly doubled among CKD patients with abnormal radionuclide stress single photon emission computed tomography (SPECT) [4, 6].

Stress cardiovascular magnetic resonance (CMR) imaging has emerged as an accurate and cost-effective modality for the diagnosis of CAD, and for risk stratification of CV events without ionizing radiation [7–9]. Several studies have shown the long-term prognostic value of both inducible ischemia and MI in patients with suspected or known CAD [7, 8, 10]. Although multiple studies have shown an incremental prognostic value of stress CMR above traditional CV risk factors in general population [7, 8], its additional prognostic value in patients with CKD remains unknown because those patients have been frequently excluded from outcome studies.

Therefore, this study aimed to determine whether detection of inducible ischemia or unrecognized MI through vasodilator stress CMR can provide incremental prognostic value above traditional CVD risk factors to predict CVD events in a cohort of patients with known CKD and without known CAD.

## Methods

### Study population

Between December 2008 and January 2021, we conducted a retrospective dual center study with enrolment of consecutive symptomatic patients with known stage 3 CKD but without known CAD, referred for vasodilator stress perfusion CMR in the Institut Cardiovasculaire Paris Sud (*ICPS, Massy, France*) and Lariboisiere University Hospital (*Assistance publique des hopitaux de Paris, APHP, Paris, France*). Known stage 3 CKD, was defined by a history of CKD in the patient’s medical record [11] including an eGFR between 30 and 60 ml/min/1.73 m^2^ assessed at least 3 months prior to the CMR exam. The chronic status of the kidney disease was also confirmed by a second eGFR assessment between 30 and 60 ml/min/1.73 m^2^ within one month prior to the CMR exam. Symptomatic patients were defined by the presence of angina or dyspnea on exertion. Following the current CMR guidelines [12], all patients with eGFR < 30 ml/min/1.73 m^2^ were excluded. In addition, all patients with known CVD were excluded, such as a known stenosis ≥ 50% in at least one epicardial coronary artery on invasive coronary angiography or coronary computed tomography angiography, patients with a positive functional test, patients with a history of revascularization (defined by previous percutaneous coronary intervention or coronary artery bypass grafting), patients with prior MI (defined by a history of acute coronary syndrome confirmed by invasive coronary angiography), history of atrial fibrillation (AF), history of peripheral atheroma, prior hospitalization for heart failure or known left ventricular (LV) dysfunction (defined by LV ejection fraction [LVEF] < 50%). The presence of symptoms (angina or dyspnea) was confirmed by a senior cardiologist on the day of stress CMR. All patients with LVEF < 50% using CMR without ischemic LGE or inducible ischemia were excluded. Other exclusion criteria are detailed in Additional file 1. To compare the prognostic values of stress CMR in patients with known stage 3 CKD versus patients without CKD, a cohort of control patients with eGFR ≥ 60 ml/min/1.73 m^2^ without known CAD was selected from our center using a propensity matching score. In addition, to assess the clinical interest of stress CMR in stage 3 CKD patients, the prognostic value of stress CMR in this cohort was compared to a control population with eGFR ≥ 60 ml/min/1.73 m^2^ from our center using a 1:1 propensity score-matched population (with eGFR between 30 and 60 ml/min/1.73 m^2^ vs with eGFR ≥ 60 ml/min/1.73 m^2^).

Clinical data were collected according to medical history and clinical examination on the day of stress CMR. All patients provided written informed consent on the day of CMR for the use of personal data for clinical research. The study was approved by the local Ethics Committee of our Institutions and conducted in accordance with the 1964 Declaration of Helsinki. This study followed the STrengthening the Reporting of OBservational Studies in Epidemiology (STROBE) reporting guideline for cohort studies.

### Patient follow-up and clinical outcomes

The follow-up consisted of a clinical visit as part of usual care (36%) or by direct contact with the subject or the referring cardiologist (64%). A clinical questionnaire with a detailed description of clinical study outcomes was filled out by senior cardiologists and radiologists. Data collection ended in January 2022. The primary outcome was the occurrence of at least one of the combined major adverse clinical events (MACE) defined as CVD mortality or nonfatal MI. The secondary outcomes were CVD mortality, all-cause mortality, and hospitalization for heart failure (HF). Clinical event adjudication was based on the follow-up clinical visit or contact, with a consensus reached by two senior cardiologists. Nonfatal MI was defined by typical angina of ≥ 20 min duration, electrocardiogram (ECG) changes, and a rise in troponin or creatine kinase level above the 99th percentile of the upper reference limit [13]. CV mortality was defined as sudden cardiac death (SCD) with documented fatal arrhythmias, or any death immediately preceded by acute MI, acute or exacerbation of HF, or stroke. All clinical events were defined according to the published standardized definitions [14], and detailed in Additional file 1: Supplemental File 2. In patients with multiple events, only the first event was considered for event-free survival analysis. According to guidelines, HF hospitalization was defined by symptoms and/or signs of HF with evidence of diastolic or systolic dysfunction by echocardiography and elevated levels of brain natriuretic peptide (BNP > 35 pg/ml and/or NT-proBNP > 125 pg/ml) [15]. In patients who underwent percutaneous coronary intervention or coronary artery bypass graft < 90 days after the index CMR examination, peri-procedural events (MI or CVD mortality, n = 9 patients) [16] were not included in the analysis.

### CMR protocol

All patients underwent CMR in dedicated CMR laboratories using 1.5 T scanners (MAGNETOM Espree, MAGNETOM Avanto or MAGNETOM Aera, Siemens Healtineers, Erlangen, Germany). The detailed CMR protocol has been previously described [17, 18] and is detailed in Additional File 1: Supplemental file 3. Briefly, long-axis and short-axis views covering the entire LV were obtained using an ECG gated balanced steady-state free-precession sequence (bSSFP). Vasodilatation was induced with dipyridamole injected at 0.84 mg/kg over 3 min for all patients in Institut Cardiovasculaire Paris Sud, and with adenosine infused at a rate of 140 mcg/kg/min over 6 min for all patients in Lariboisiere University Hospital. At the end of vasodilator agent infusion, a bolus of gadolinium-based contrast agent (Dotarem, Guerbet, Aulnay-sous-Bois, France, 0.1 mmol/kg) was injected at a rate of 5.0 ml/s. Stress perfusion imaging was performed using a saturation-prepared ECG gated bSSFP sequence with the following typical parameters: repetition time/echo time = 287/1.2 ms, acceleration factor = 2, field of view = 370 × 314 mm, reconstructed pixel size = 1.7 × 1.7 × 8 mm. A series of six slices (four short-axis views, in addition to 2- and 4-chamber views) were acquired every other heartbeat. Then, theophylline was injected intravenously (250 mg over 5 min) to null the effect of dipyridamole. Ten minutes after contrast injection, single-breath-hold 3D T1-weighted inversion-recovery gradient-echo sequence was acquired with the same prescriptions to detect late gadolinium enhancement (LGE). The inversion time was individually adjusted to null normal myocardium. In case of artifacts on LGE images, additional 2D single-shot bSSFP images with phase sensitive inversion recovery reconstruction were acquired. CMR sequence parameters are detailed in Additional File 1: Supplemental File 4. Patients were asked to refrain from caffeine at least 12 h before CMR. Safety was studied with clinical monitoring one hour after CMR to assess major adverse events. A 12-lead ECG was performed both before and after CMR examination.

### CMR analysis

LV end-diastolc and end-systolc volumes and LVEF were calculated from the short-axis cine images (Syngo.via, Siemens Healthineers). Stress perfusion and LGE images were evaluated according to the 17-segment model of the American Heart Association [19]. The analysis of perfusion images was done visually by two experienced physicians in each center blinded to clinical and follow-up data. Inducible ischemia was defined as a subendocardial perfusion defect that (1) occurred in at least one myocardial segment affecting at least two different views (short-axis and long-axis views), (2) persisted for at least three phases beyond peak contrast enhancement, (3) followed a coronary distribution, and (4) occurred in the absence of co-localized LGE in the same segment [20–23]. An unrecognized MI was defined by LGE with ischemic pattern defined by subendocardial or transmural LGE, without the use of any clinical or ECG data [24]. As previously described [25], the total number of ischemic segments was measured using a semi-quantitively method for each patient. Mild, moderate, and severe ischemia were defined as the involvement of 1 to 2, 3 to 5, and ≥ 6 myocardial segments, respectively. LGE was semi-quantitatively assessed using the number of LGE segments. Using a random sample size of 50 patients, inter-observer differences regarding the identification of ischemia were negligible (kappa coefficient: 0.91 (95% CI 0.87 to 0.94). All clinical and CMR characteristics were reported in a dedicated database (Hemolia, Clinigrid Inc., Paris, France for Institut Cardiovasculaire Paris Sud; and Middlecare Inc., Evolucare, Paris, France for Lariboisiere University Hospital).

### Statistical analysis

Continuous variables were expressed as mean ± standard deviation (SD) and categorical variables as frequency with percentage. Follow-up was presented as median and interquartile range (IQR). Differences between patients with and without ischemia in terms of baseline clinical and CMR characteristics were compared using the Student’s t-test or the Wilcoxon rank-sum test for continuous variables and the chi-square or Fisher’s exact test for categorical variables, as appropriate. Normal distribution was assessed using the Shapiro–Wilk test. Cumulative incidence rates of individual and composite outcomes were estimated using the Kaplan–Meier method and compared with the log-rank test. The proportional hazard assumption was visually assessed using Schoenfeld residuals. Data on patients who were lost to follow-up were censored at the time of the last contact. Cox proportional hazards methods were used to identify the predictors of MACE among patients with or without inducible ischemia, and with or without unrecognized MI. The assumption of proportional hazards ratio (HR) was verified.

The different multivariable models used for adjustment were as follows:

**Model 1:** included traditional CV risk factors with age, male sex, BMI, diabetes mellitus, hypertension, dyslipidemia, current or previous smoking, family history of CAD, LVEF, eGFR, and time between CKD diagnosis and CMR exam.
**Model 2:** model 1 + presence of unrecognized MI.
**Model 3:** model 2 + presence of ischemia.
**Model 4:** model 2 + number of segments with inducible ischemia.

The discriminative capacity of each model for predicting MACE was determined according to the Harrell’s C-statistic at baseline and after addition of ischemia and unrecognized MI. The additional predictive value of the presence of ischemia and MI was calculated by the Harrell’s C-statistic increment, the continuous net reclassification improvement (NRI), and the integrative discrimination index (IDI) [26]. NRI and IDI were computed at the end of follow-up using the R package “survIDINRI” [27].

To assess the clinical value of stress CMR in stage 3 CKD patients, the prognostic value of stress CMR in this cohort was compared to a control population with eGFR ≥ 60 ml/min/1.73 m^2^ from our center using a 1:1 propensity score-matched population (with eGFR between 30 and 60 ml/min/1.73 m^2^ vs with eGFR ≥ 60 ml/min/1.73 m^2^). A multivariable logistic regression model was built to estimate a propensity score for known CKD, using the following variables: age, gender and traditional cardiovascular risk factors. Imbalances between groups were considered to be small when the absolute standardized difference for a given covariate was less than 10%. The probit model with 1-to-1 nearest neighbor matching and without replacement was used to identify one patient with eGFR between 30 and 60 ml/min/1.73 m^2^ (N = 702) for each patient with eGFR ≥ 60 ml/min/1.73 m^2^ (N = 702). The association between the presence of ischemia and the occurrence of MACE in the matched groups was assessed using a Cox proportional hazards regression model.

The prognostic value of ischemia in different subsamples of clinical interest was investigated by a Forest plot. A sensitivity analysis of the prognostic value of ischemia stratified by center was also performed. A two-tailed p-value < 0.05 was considered statistically significant. Statistical analysis was performed using R software (version 3.3.1, R Project for Statistical Computing, Vienna, Austria).

## Results

### Patient characteristics

**Fig. 1:**
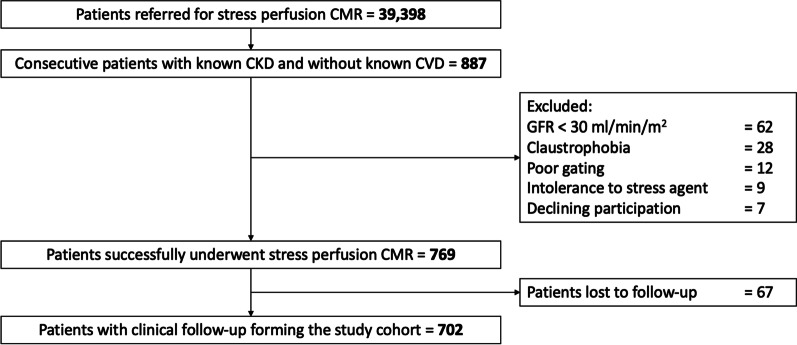
Study flowchart. *CKD* chronic kidney disease; *CMR* cardiovascular magnetic resonance; *CVD* cardiovascular disease; *ECG* electrocardiogram

From the initial cohort of 39,398 consecutive patients referred for stress CMR, 887 (2.3%) had known CKD (652 patients in Institut Cardiovasculaire Paris Sud and 235 patients in Lariboisiere Hospital), and 769 (86.7%) patients successfully completed the stress CMR examination. Reasons for failure to complete CMR are detailed in the study flowchart (Fig. 1). Overall, 702 patients (91.3%) had clinical follow-up and constituted the study cohort. Among those, 618 (88.0%) patients had symptomatic angina and 88 (12.0%) patients had dyspnea on exertion.

**Fig. 2:**
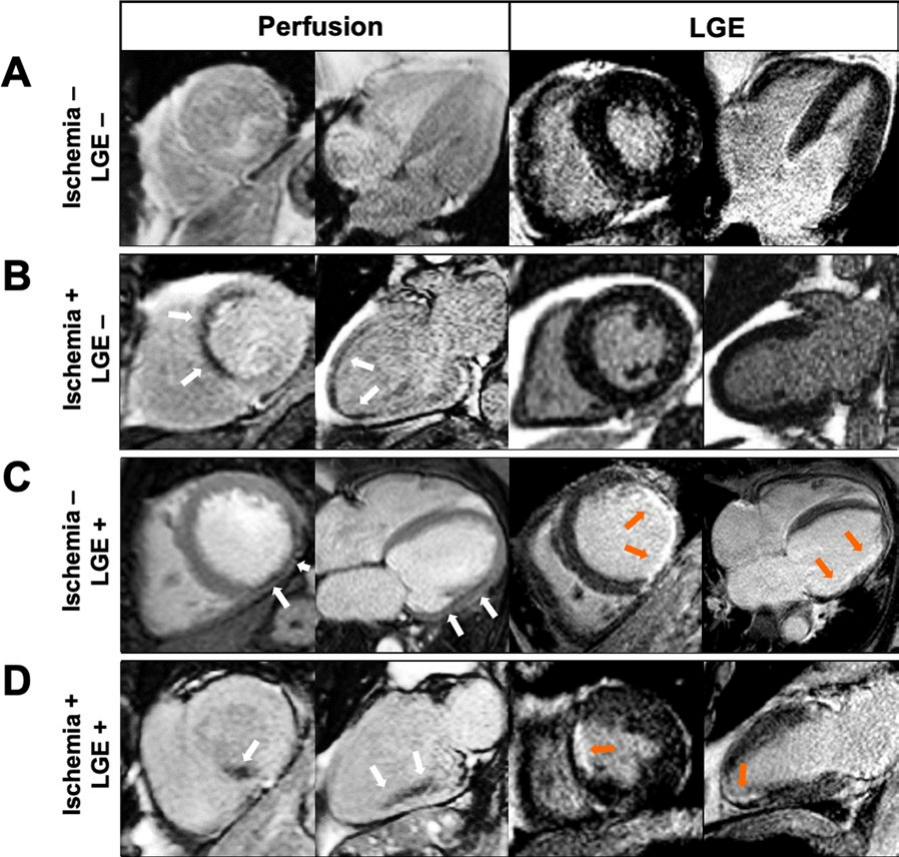
Examples of inducible myocardial ischemia on stress CMR in patients with known CKD. **A** normal. 77-year-old male with hypertension and history of CKD (GFR 38 ml/min/m^2^), presenting atypical angina. Stress CMR revealed no perfusion defect and LGE was negative, ruling out the diagnosis of myocardial ischemia. **B** Inducible ischemia. 69-year-old female with and history of CKD (GFR 56 ml/min/m^2^), presenting dyspnea on exertion. First-pass myocardial stress perfusion images revealed a reversible perfusion defect of the anteroseptal wall (*white arrows*) without LGE, indicative of myocardial inducible ischemia suggestive of significant LAD stenosis, confirmed by coronary angiography. **C** Myocardial scar without ischemia. 70-year-old female with diabetes mellitus, hypertension and history of CKD (GFR 41 ml/min/m^2^), presenting dyspnea on exertion. Stress CMR showed a subendocardial lateral scar on LGE (*orange arrows*), with a colocalization of the perfusion defect (*white arrows*) and, therefore, no inducible ischemia. Coronary angiography confirmed the absence of significant stenosis. **D **Myocardial scar with additional inducible ischemia. 67-year-old male with diabetes mellitus, hypertension and history of CKD (GFR 55 ml/min/m^2^), presenting atypical angina. Stress CMR showed a subendocardial scar on the antero-septo-apical wall on LGE sequences (*orange arrows*), and a perfusion defect of the inferior and infero-septal wall (*white arrows*) on first-pass perfusion images, indicative of inducible myocardial ischemia. Coronary angiography revealed high-grade stenoses of the RCA. *CAD* coronary artery disease; *CMR* cardiovascular magnetic resonance; *Cx* circumflex coronary artery; *LAD* left anterior descending; *LGE* late gadolinium enhancement; *MI* myocardial infarction; *NSTEMI* non-ST segment elevation myocardial infarction; *PCI* percutaneous coronary intervention; *RCA* right coronary artery; *STEMI* ST segment elevation myocardial infarction

Baseline patient characteristics and CMR findings stratified by the presence of inducible ischemia are shown in Table 1. The mean age of the study population was 71.4 ± 8.8 years. Seventy percent of patients were male, 75% had hypertension, 59% dyslipidemia, 49% diabetes mellitus, 15% were current or previous smokers, and 8% had a family history of CAD. The mean eGFR was 41 ± 9 ml/min/1.73 m^2^. In the overall population, 15% of patients had 2 CVD risk factors, 39% had 3, and 46% had ≥ 4 CVD risk factors. The mean LVEF was 62.8 ± 9.8%. Inducible ischemia was detected in 145 patients (20.7%) and unrecognized MI in 112 (16.0%). Twenty-six patients (3.7%) had both ischemia and unrecognized MI. A total of 218 (31.1%) patients had an abnormal CMR exam, defined by the presence of ischemia and/or unrecognized MI (Fig. 2). Patients with ischemia had more diabetes (63.5% vs. 45.8%) and had a higher 10-year risk of fatal CAD (4.2% vs. 3.5%, both p < 0.001) than patients without ischemia. Other CAD risk factors including hypertension, dyslipidemia, and smoking, were similar between the two groups.

**Table 1:** Baseline clinical and cardiovascular magnetic resonance (CMR) characteristics of stage 3 chronic kidney disease (CKD) patients with or without inducible ischemia (N = 702)

	All patients (N = 702)	Without ischemia (N = 557)	With ischemia (N = 145)	p value
Demographics				
Age, years	71.4 ± 8.8	71.3 ± 8.8	71.7 ± 8.8	0.64
Male, n (%)	492 (70.1)	392 (70.4)	100 (69.0)	0.46
BMI, kg/m^2^	26.8 ± 3.2	27.0 ± 3.3	26.0 ± 2.6	** < 0.001**
Cardiovascular risk factors, n (%)				
Diabetes mellitus	347 (49.4)	255 (45.8)	92 (63.5)	** < 0.001**
Hypertension	525 (74.8)	413 (74.1)	112 (77.2)	0.51
Dyslipidemia	411 (58.5)	326 (58.5)	85 (58.6)	1.00
Current or previous smoking	107 (15.2)	86 (15.4)	21 (14.5)	0.88
Family history of CAD	54 (7.7)	51 (9.2)	3 (2.1)	**0.007**
Obesity (BMI ≥ 30 kg/m^2^)	84 (12.0)	80 (14.4)	4 (2.8)	** < 0.001**
Time between CKD diagnosis and CMR, years	3.6 ± 2.9	3.2 ± 2.8	5.4 ± 3.1	** < 0.001**
eGFR, ml/min/1.73 m^2^	41 ± 9	43 ± 9	36 ± 10	** < 0.001**
Ten-year risk for fatal CAD (%)*	3.6 (1.0–5.9)	3.5 (0.9–5.8)	4.2 (1.2–6.3)	** < 0.001**
Indications to stress CMR, n (%)				
Symptomatic angina	618 (88.0)	513 (92.1)	105 (72.4)	** < 0.001**
Dyspnea	88 (12.0)	44 (7.9)	44 (30.3)	** < 0.001**
Stress CMR findings				
LV ejection fraction, %	62.8 ± 9.8	62.8 ± 9.1	62.8 ± 12.1	0.97
LV end-diastolic volume index, ml/m^2^	62.8 ± 13.4	62.5 ± 12.9	63.9 ± 16.2	0.34
LV end-systolic volume index, ml/m^2^	22.9 ± 4.9	23.0 ± 4.9	22.4 ± 4.7	0.20
LV mass, g/m^2^	89 ± 12	89 ± 11	90 ± 12	0.41
RV dysfunction^†^	14 (1.9)	10 (1.8)	4 (2.8)	0.13
Presence of unrecognized MI, n (%)	112 (16.0)	77 (13.8)	35 (24.1)	**0.004**
Number of segments of LGE	0.4 ± 1.1	0.3 ± 0.8	0.9 ± 1.8	** < 0.001**
Number of segments of ischemia	0.5 ± 1.3	0.0 ± 0.0	2.4 ± 1.7	** < 0.001**
RPP at baseline, mmHg/beats/min	9.1 (7.0–11.5)	9.0 (6.8–11.4)	9.3 (7.1–11.7)	0.65
RPP at stress, mmHg/beats/min	10.8 (8.4–13.0)	10.8 (8.6–12.9)	11.0 (9.2–13.6)	0.70
Recruitment center, n (%)				
Institut cardiovasculaire Paris Sud	513 (73.1)	394 (70.7)	119 (82.1)	** < 0.001**
Lariboisiere University Hospital	189 (26.9)	163 (29.3)	26 (17.9)	** < 0.001**

Values are n (%), mean ± SD, or median (interquartile range)

*BMI* body mass index; *CMR* cardiovascular magnetic resonance; *eGFR* estimated glomerular filtration rate; *LGE* late gadolinium enhancement; *LV* left ventricle; *MI* Myocardial infarction; *RPP* rate-pressure product (pressure mmHg × heart rate bpm)/1000; *SD*: standard deviation; *RV* right ventricle

*Based on a modified SCORE project (https://www.escardio.org/Education/Practice-Tools/CVD-prevention-toolbox/SCORE-Risk-Charts) that did not take into account the total cholesterol level

^†^Defined by right ventricle ejection fraction < 45%

Using the propensity score-matched populations (with eGFR between 30 and 60 ml/min/1.73 m^2^ vs with eGFR ≥ 60 ml/min/1.73 m^2^), stage 3 CKD patients had a higher rate of both ischemia (20.7 vs. 15.7%) and unrecognized MI (16.0 vs. 14.0%, both p < 0.001) than patients with eGFR ≥ 60 ml/min/1.73 m^2^ (Table 2)*.*

**Table 2:** Baseline and CMR characteristics of the propensity-matched population and of patients with stage 3 CKD and patients with eGFR ≥ 60 ml/min/1.73 m^2^

	Patients with stage 3 CKD (N = 702)	Propensity-matched patients with eGFR ≥ 60 ml/min/1.73 m^2^ (N = 702)	p value
Demographics			
Age, years	71.4 ± 8.8	71.3 ± 8.7	0.63
Male, n (%)	492 (70.1)	299 (55.2)	1.000
BMI, kg/m^2^	26.8 ± 3.2	27.3 ± 3.7	** < 0.001**
Coronary risk factors, n (%)			
Diabetes mellitus	260 (48.0)	260 (48.0)	1.000
Hypertension	402 (74.2)	402 (74.2)	1.000
Dyslipidemia	316 (58.3)	315 (58.1)	0.91
Current or previous smoking	177 (32.7)	177 (32.7)	1.000
Family history of CAD	45 (8.3)	42 (7.7)	0.78
Ten-year risk for fatal CAD (%)*	2.4 (0.8–5.6)	2.4 (0.8–5.7)	0.81
Stress CMR			
LV ejection fraction, %	62.7 ± 10.0	68.9 ± 11.2	** < 0.001**
LV end-diastolic volume index, ml/m^2^	62.7 ± 13.6	60.1 ± 12.3	** < 0.001**
LV end-systolic volume index, ml/m^2^	23.0 ± 5.2	24.1 ± 5.0	0.08
Presence of unrecognized MI, n (%)	91 (16.8)	66 (12.2)	** < 0.001**
Number of segments of LGE	0.4 ± 1.1	0.2 ± 1.0	** < 0.001**
Presence of ischemia	97 (17.9)	76 (14.0)	** < 0.001**
Number of segments of ischemia	0.4 ± 1.0	0.3 ± 0.9	** < 0.001**
RPP at baseline, mmHg/beats/min	9.1 (7.0–11.3)	8.9 (6.7–11.0)	** < 0.001**
RPP at stress, mmHg/beats/min	10.5 (8.1–12.6)	10.2 (7.6–12.5)	** < 0.001**

Values are n (%), mean ± SD, or median (interquartile range)

*BMI* body mass index; *CAD* coronary artery disease; *CMR* cardiovascular magnetic resonance; *LGE* late gadolinium enhancement; *LV* left ventricle; *MI* Myocardial infarction; *RPP* rate-pressure product (pressure mmHg × Heart rate bpm)/1000; *SD* standard deviation; *RV* right ventricle

*Based on a modified SCORE project (https://www.escardio.org/Education/Practice-Tools/CVD-prevention-toolbox/SCORE-Risk-Charts) that did not take into account the total cholesterol level

### Safety results

Detailed safety results are presented in Additional File 1: Supplemental File 5. No patient died during or shortly after CMR and there were three cases of unstable angina. No complication related to the injection of gadolinium or nephrogenic systemic fibrosis case have been reported.

### Primary outcome

**Fig. 3:**
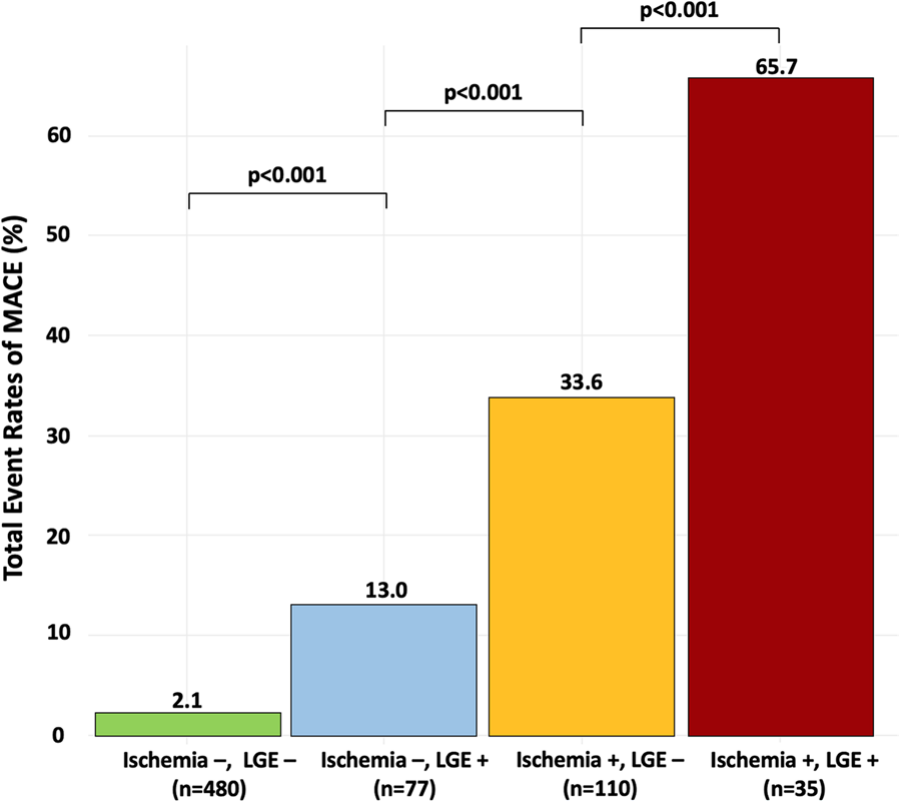
Cumulative rates of MACE during follow-up stratified by the presence or absence of inducible ischemia and by the presence or absence of unrecognized myocardial infarction (MI)

**Fig. 4:**
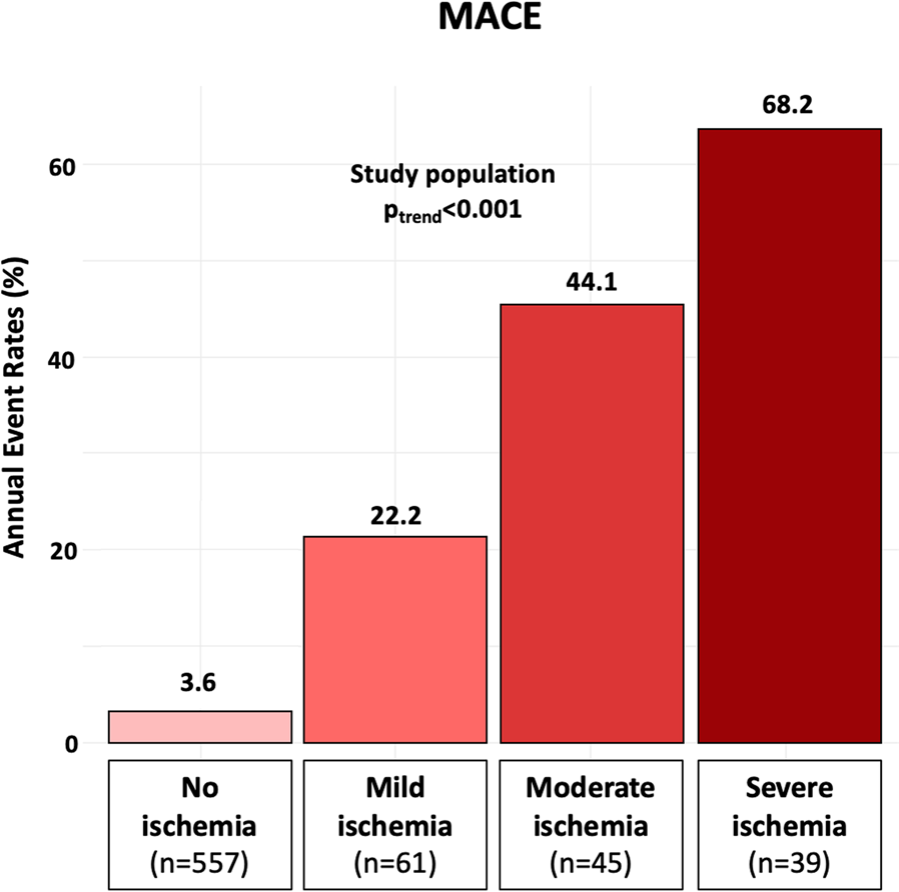
Cumulative rates of MACE during follow-up stratified by the extent of inducible ischemia. Mild, moderate, and severe ischemia were defined as the involvement of 1 to 2, 3 to 5, and ≥ 6 myocardial segments, respectively. Comparison tests were based on the Cochran-Armitage test for trend

The median (interquartile range; IQR) follow-up duration was 6.4 (4.0–8.2) years. Of the 702 patients, 80 (11.4%) experienced a MACE, including 48 (6.8%) CVD deaths and 32 nonfatal MI (4.6%). The annualized rate of MACE was 3.6%/year. The total event rates for MACE according to the presence or absence of myocardial ischemia and unrecognized MI are presented in Fig. 3. Patients without myocardial ischemia or unrecognized MI had a lower rate of MACE (2%), whereas the cumulative rate of MACE was greater for patients with both myocardial ischemia and unrecognized MI (66%, p < 0.001). Patients with ischemia but without unrecognized MI had higher cumulative rate of MACE (34%) than patients with unrecognized MI but without ischemia (13%, p < 0.001). The rate of MACE increased with the ischemic burden assessed by the number of ischemic segments (p-trend < 0.001, Fig. 4).

### Prognostic factors of outcomes

In univariable analysis, diabetes mellitus, LVEF, presence and extent of ischemia, presence and extent of unrecognized MI were all significantly associated with MACE (Table 3). Using Kaplan–Meier analysis, the presence of inducible ischemia and unrecognized MI were associated with the occurrence of MACE (HR 12.5; 95% CI 7.50 to 20.8; and HR 4.31; 95% CI 2.74 to 6.79; both p < 0.001; respectively) (Fig. 4). These findings were similar in both centers Institut Cardiovasculaire Paris Sud and Lariboisiere University Hospital (HR 12.7; 95% CI 6.82 to 23.5; and HR 12.60; 95% CI 4.97 to 32.1; both p < 0.001; respectively; Additional File 1: Supplemental File 6).

**Table 3:** Univariable analysis of clinical and CMR characteristics for prediction of MACE

	MACE		Cardiovascular Mortality	
	Hazard Ratio (95% CI)	p value	Hazard Ratio (95% CI)	p value
Age	1.02 (0.99–1.05)	0.14	1.08 (1.03–1.12)	**0.001**
Male	1.15 (0.68–1.93)	0.61	0.68 (0.37–1.26)	0.22
BMI	1.03 (0.96–1.10)	0.41	0.98 (0.89–1.07)	0.60
Diabetes mellitus	4.39 (2.49–7.71)	** < 0.001**	4.87 (2.27–10.5)	** < 0.001**
Hypertension	1.44 (0.82–2.53)	0.21	1.94 (0.87–4.35)	0.11
Dyslipidemia	0.66 (0.42–1.03)	0.07	0.56 (0.31–1.01)	0.05
Current or previous smoking	1.61 (0.94–2.75)	0.09	1.05 (0.47–2.35)	0.91
Family history of CAD	1.03 (0.45–2.38)	0.94	1.92 (0.81–4.53)	0.14
eGFR, per 10 units	0.73 (0.50–0.83)	**0.012**	0.53 (0.37–0.68)	** < 0.001**
Time between CKD diagnosis and CMR exam	1.62 (0.91–2.59)	0.19	1.99 (0.91–4.40)	0.10
Dyspnea	2.68 (1.60–4.47)	** < 0.001**	6.07 (3.36–11.0)	** < 0.001**
LVEF, per 10%	0.88 (0.72–1.10)	0.28	1.22 (0.88–1.70)	0.24
LV end-diastolic volume index, per 10 ml/m^2^	0.96 (0.81–1.14)	0.64	0.86 (0.68–1.08)	0.19
LV end-systolic volume index, per 10 ml/m^2^	0.78 (0.50–1.21)	0.27	0.44 (0.25–0.73)	**0.002**
Presence of unrecognized MI	4.31 (2.74–6.79)	** < 0.001**	4.27 (2.35–7.75)	** < 0.001**
Number of segments with unrecognized MI	2.09 (1.88–2.33)	** < 0.001**	2.15 (1.87–2.47)	** < 0.001**
Presence of inducible ischemia	12.5 (7.50–20.8)	** < 0.001**	9.76 (5.19–18.4)	** < 0.001**
Number of segments with ischemia	1.68 (1.55–1.83)	** < 0.001**	1.58 (1.40–1.77)	** < 0.001**

Same as Table 1

*BMI* body mass index; *CAD* coronary artery disease; *CI* confidence interval; *LV* Left ventricle; *LVEF* left ventricular ejection fraction; *MACE* major adverse cardiac events; *MI* myocardial infarction

**Fig. 5:**
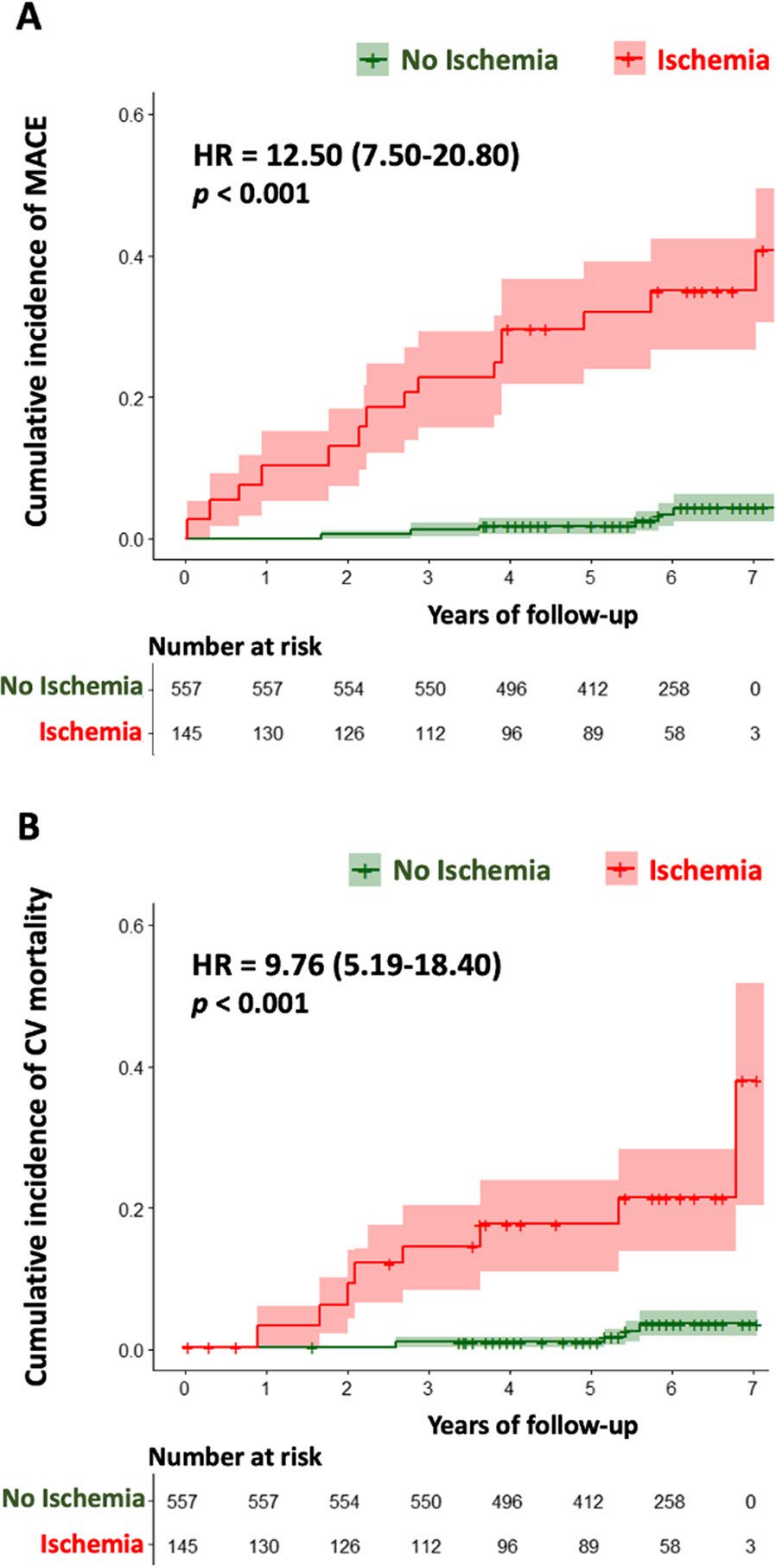
Kaplan–Meier curves for MACE (**A**) and Cardiovascular mortality (**B**) stratified by the presence of inducible ischemia. Test comparing the two groups is based on the log-rank test

In addition, ischemia was also associated with CVD mortality (HR 9.76; 95% CI 5.19 to 18.4; p < 0.001), nonfatal MI (HR 16.9; 95% CI 6.92 to 37.3; p < 0.001), and all-cause of mortality (HR 2.56; 95% CI 1.70 to 3.86; p < 0.001) (Additional File 1: Supplemental File 7). The prognostic value of ischemia remained consistent in all other subsamples of clinical interest such as men and women, diabetics and non-diabetics, and regardless of LVEF (Fig. 5).

### Independent prognostic value of ischemia and unrecognized MI

**Fig. 6:**
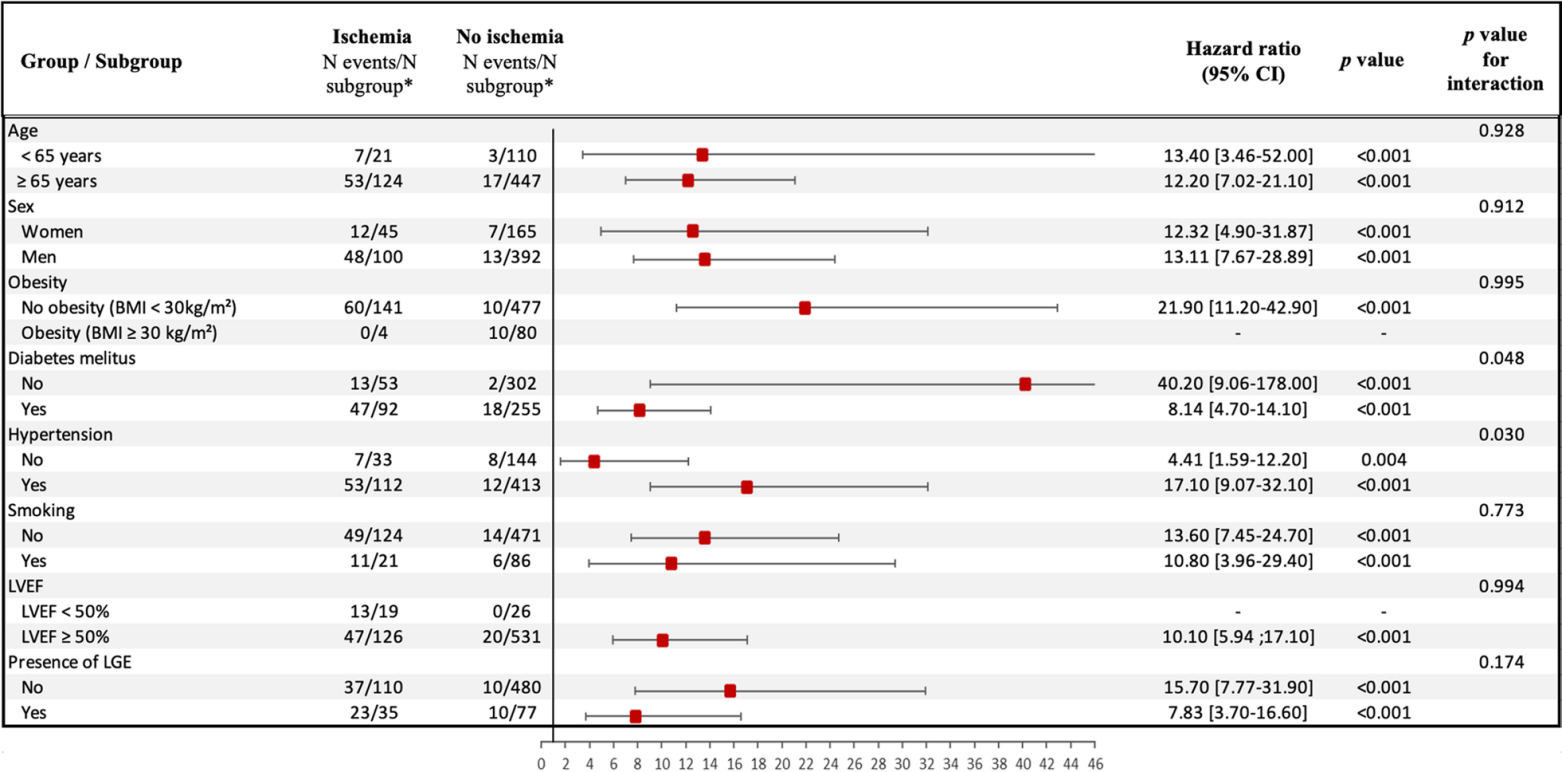
Subgroup analysis. Forest-plot of incidence of MACE based on the presence of silent ischemia in prespecified subgroups. *N events/N subgroup: number of patients who had a major adverse clinical event (MACE)/number of patients in the subgroup

In multivariable stepwise Cox regression (model 3), the presence of ischemia and unrecognized MI were independent predictors of a higher incidence of MACE (HR = 15.5; 95% CI 7.72 to 30.9, and HR = 4.67; 95% CI 2.83 to 7.68, both p < 0.001; respectively) and CVD mortality (HR = 7.67; 95% CI 2.61 to 22.6, and HR = 6.21; 95% CI 3.10 to 12.5, both p < 0.001; respectively). After adjustment, the extent of ischemia was also independently associated with MACE (HR = 1.19; 95% CI 1.10 to 1.29, p < 0.001) and CV mortality (HR = 1.11; 95% CI 1.04 to 1.52, p = 0.021) (Table 4). In competing risk analysis, the presence of ischemia was independently associated with nonfatal MI and CVD mortality (both p < 0.001) (Table 5 and Fig. 6).

**Table 4:** Multivariable Cox regression analysis for the prediction of adverse events

	MACE		Cardiovascular mortality	
	Hazard ratio (95% CI)	p value	Hazard ratio (95% CI)	p value
Model 1*				
Age	1.02 (0.99–1.06)	0.17	1.09 (1.05–1.15)	** < 0.001**
Male	0.86 (0.51–1.48)	0.59	0.80 (0.48–1.46)	0.67
BMI	1.05 (0.99–1.13)	0.12	1.04 (0.95–1.14)	0.397
Diabetes mellitus	5.22 (0.88–9.50)	** < 0.001**	8.27 (3.59–19.1)	** < 0.001**
Hypertension	3.66 (1.99–6.73)	** < 0.001**	5.50 (2.26–13.4)	** < 0.001**
Dyslipidemia	0.77 (0.51–1.25)	0.63	0.59 (0.30–1.08)	0.09
Current or previous smoking	2.81 (1.55–5.11)	** < 0.001**	1.36 (0.62–3.09)	0.28
Family history of CAD	1.08 (0.47–2.46)	0.78	1.90 (0.78–4.50)	0.35
LVEF	0.92 (0.72–1.19)	0.54	0.87 (0.66–1.22)	0.68
eGFR	0.83 (0.61–0.97)	**0.03**	0.71 (0.52–0.89)	**0.02**
Time between CKD diagnosis and CMR exam	1.62 (0.91–2.59)	0.19	1.99 (0.91–4.40)	0.10
Model 2^†^				
Presence of unrecognized MI	5.07 (3.13–8.21)	** < 0.001**	6.15 (2.98–12.1)	** < 0.001**
Model 2bis^†^				
Presence of inducible ischemia	16.4 (8.31–34.2)	** < 0.001**	8.22 (3.08–26.2)	** < 0.001**
Model 3^‡^				
Presence of unrecognized MI	4.67 (2.83–7.68)	** < 0.001**	6.21 (3.10–12.5)	** < 0.001**
Presence of inducible ischemia	15.5 (7.72–30.9)	** < 0.001**	7.67 (2.61–22.6)	** < 0.001**
Model 4^§^				
Presence of unrecognized MI	4.60 (2.80–7.66)	** < 0.001**	6.02 (2.89–11.8)	** < 0.001**
Number of segments of inducible ischemia	1.19 (1.10–1.29)	** < 0.001**	1.11 (1.04–1.52)	**0.021**

*Model 1 included traditional CV risk factors: age, male, BMI, diabetes mellitus, hypertension, dyslipidemia, current or previous smoking, family history of CAD, LVEF, eGFR and time between CKD diagnosis and CMR exam

^†^Model 2 included: model 1 + unrecognized MI or inducible ischemia (2 bis)

^‡^Model 3 included: model 2 + inducible ischemia

^§^Model 4 included: model 2 + number of segments with inducible ischemia

*BMI* body mass index; *CAD* coronary artery disease; *CI* confidence interval, *CV* cardiovascular; *LGE* late gadolinium enhancement; *LVEF* left ventricular ejection fraction; *MACE* major

**Table 5:** Univariable and multivariable competing risk regression analysis (N = 702)

	Nonfatal MI				Cardiovascular mortality			
	Univariable analysis		Multivariable analysis^†^		Univariable analysis		Multivariable analysis^†^	
	sHR* (95% CI)	p value	sHR* (95% CI)	p value	sHR* (95% CI)	p value	sHR* (95% CI)	p value
Age	0.97 (0.95–0.99)	**0.009**	1.08 (1.03–1.13)	**0.002**	1.08 (1.03;1.12)	** < 0.001**	1.09 (1.03–1.15)	** < 0.001**
Male	3.64 (1.11–11.9)	**0.033**	1.02 (0.99–1.06)	0.23	0.66 (0.36–1.21)	0.18	-	-
BMI	1.10 (1.05–1.16)	** < 0.001**	1.16 (1.06–1.27)	**0.001**	0.97 (0.89–1.07)	0.55	-	-
Diabetes mellitus	3.73 (1.61–8.62)	**0.002**	4.61 (1.89–11.2)	** < 0.001**	4.69 (2.19: 10.00)	** < 0.001**	5.30 (1.78–11.3)	** < 0.001**
Hypertension	0.99 (0.45–2.19)	0.98	-	-	1.93 (0.87–4.27)	0.11	1.99 (0.85–4.43)	0.72
Dyslipidemia	0.83 (0.40–1.74)	0.64	-	-	0.57 (0.32–1.01)	0.055	-	-
Current or previous smoking	2.55 (1.20–5.43)	**0.015**	2.30 (0.87–7.26)	0.41	0.99 (0.44–2.22)	0.98	-	-
LVEF	0.96 (0.93–0.99)	**0.002**	0.97 (0.92–1.06)	0.57	1.02 (0.99–1.06)	0.23	-	-
eGFR	0.80 (0.59–0.95)	**0.02**	0.87 (0.71–1.04)	0.41	0.70 (0.50–0.87)	**0.003**	0.78 (0.62–0.98)	**0.04**
Time between CKD diagnosis and CMR exam	1.01 (0.97–1.05)	0.58	-	-	1.89 (0.87–4.23)	0.45	-	-
Presence of inducible ischemia	17.0 (7.09–41.0)	** < 0.001**	8.21 (4.22–15.9)	** < 0.001**	8.49 (4.53–15.9)	** < 0.001**	7.35 (4.02–13.9)	** < 0.001**
Presence of unrecognized MI	4.08 (1.98–8.42)	** < 0.001**	5.31 (2.75–10.2)	** < 0.001**	3.79 (2.10–6.82)	** < 0.001**	6.02 (2.97–11.9)	** < 0.001**

*HR of the subdistribution hazard function

^†^Covariates by stepwise variable selection with entry and exit criteria set at the p < 0.20 level: for nonfatal MI: age, male, body mass index, diabetes mellitus, smoking, LVEF, eGFR, presence of unrecognized MI, and presence of ischemia; for CV mortality: age, diabetes mellitus, hypertension, eGFR, presence of unrecognized MI, and presence of ischemia

Same as in Table 2. *HR* hazard ratio

Using propensity score-matching, the prognostic value of ischemia (HR = 15.5; 95% CI 7.72 to 30.9 versus HR = 3.74; 95% CI 2.77 to 5.26, p < 0.001) and unrecognized MI (HR = 4.67; 95% CI 2.83 to 7.68 versus HR = 1.70; 95% CI 1.22 to 2.51, p < 0.001) to predict the occurrence of MACE were greater in stage 3 CKD patients compared to patients with eGFR ≥ 60 ml/min/1.73 m^2^ (Additional File 1: Supplemental File 8).

### Incremental prognostic value of ischemia and unrecognized MI

For the prediction of MACE, C-statistic values were 0.74 (95% CI 0.69 to 0.78) for “model 1” with traditional risk factors. The addition of unrecognized MI significantly improved the C-statistic value to 0.82 (95% CI 0.76 to 0.87; C-statistic improvement for “model 1”: 0.08; NRI = 0.250; IDI = 0.035; all p < 0.001). The addition of both unrecognized MI and ischemia significantly improved the C-statistic value to 0.87 (95% CI 0.83 to 0.90; C-statistic improvement for “model 1”: 0.13; NRI = 0.477; IDI = 0.049; all p < 0.001) (Table 6).

**Table 6:** Discrimination and reclassification associated with in inducible ischemia and unrecognized MI for the prediction of MACE

	MACE		
	C-index (95%CI)	NRI (95%CI)	IDI (95%CI)
Model 1 (traditional CV risk factors) *	0.74 (0.69–0.78)	Reference	Reference
Model 2 (model 1 + unrecognized MI) ^†^	0.82 (0.76–0.87)	0.250 (0.067–0.440)	0.035 (0.018–0.060)
Model 3 (model 2 + inducible ischemia) ^‡^	0.87 (0.83–0.90)	0.477 (0.236–0.678)	0.049 (0.025–0.071)

*Model 1 included traditional CV risk factors: age, male, BMI, diabetes mellitus, hypertension, dyslipidemia, current or previous smoking, family history of CAD, LVEF, GFR, and time between CKD diagnosis and CMR exam

^†^**Model 2** included: model 1 with unrecognized MI

^‡^**Model 3** included: model 2 with inducible ischemia

Same as in Table 2. *IDI* integrative discrimination index; *NRI* net reclassification improvement

## Discussion

**Fig. 7:**
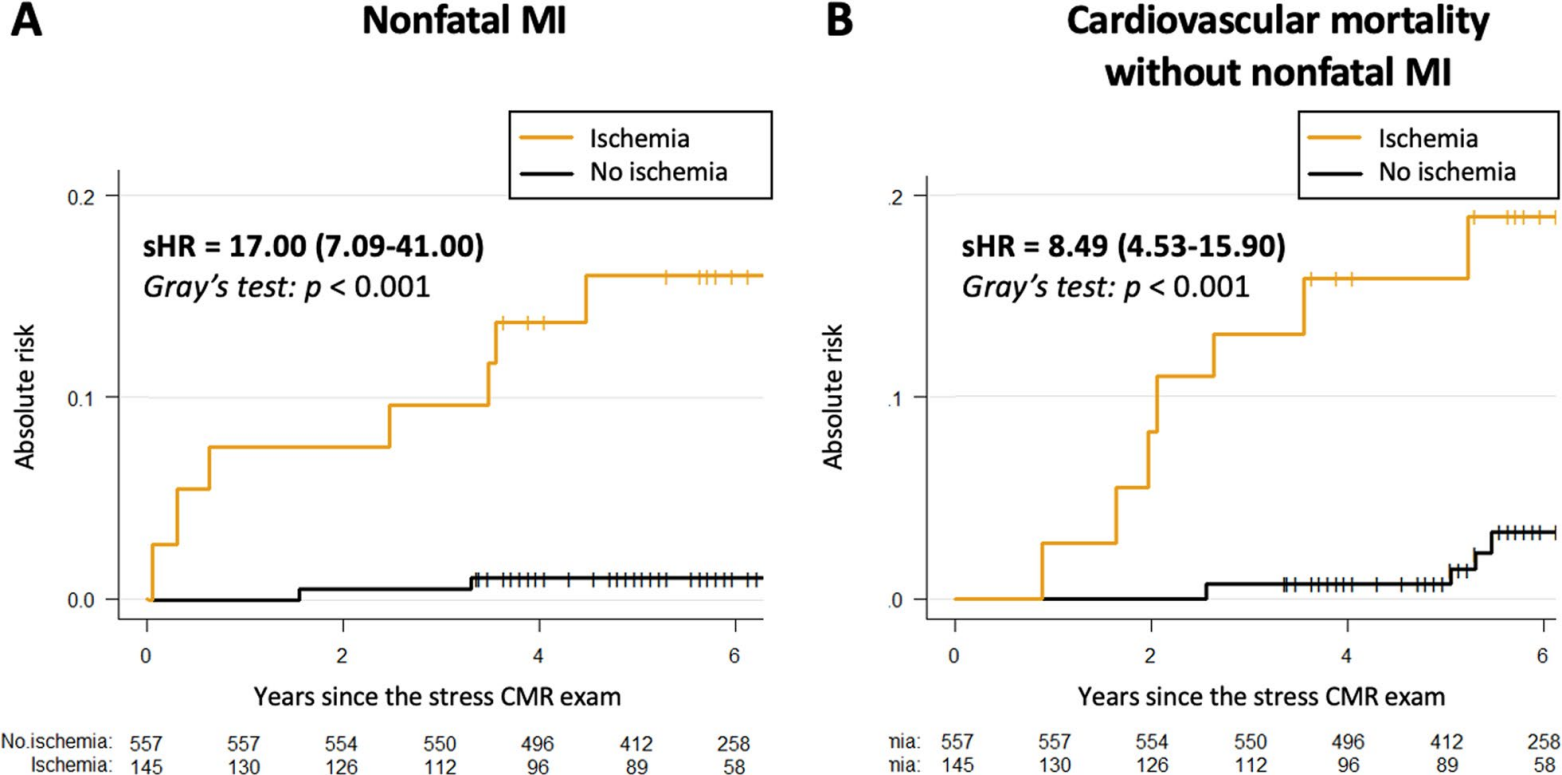
Competing risk analysis. Cumulative incidence functions of nonfatal MI (**A**) and cardiovascular mortality without nonfatal MI (**B**)

In a population of symptomatic patients with known stage 3 CKD but without known CAD referred for stress CMR, the main findings are: (1) 21% of patients had inducible ischemia and 16% had unrecognized MI; (2) in the overall population, 11.4% had MACE after median follow-up of 6 years; (3) both ischemia and unrecognized MI were independent long-term predictors of MACE and CVD mortality; (4) the extent of ischemia was also independently associated with MACE and CVD mortality; and (5) stress CMR findings, including the presence of ischemia and unrecognized MI, improved model discrimination and reclassification for the prediction of MACE above traditional risk factors (Fig. 7).

### Prevalence of CAD in patients with known CKD

In our population of patients without known CAD, the prevalence of CAD was substantial with 31% of patients having ischemia or unrecognized MI. This finding is in line with several cohorts of patients with known CKD, in which the prevalence of obstructive CAD was approximately one-third of patients [4]. Consistently, as eGFR declines below 60 to 75 ml/min/1.73 m^2^, the probability of developing CAD increases linearly [4]. In the current study, the prevalence of ischemia (21%) was in line with recent stress CMR studies in symptomatic patients that reported a prevalence of 17–26% in patients without known CAD [7, 8]. Similar to the Stress CMR Perfusion Imaging in the United States (SPINS) study, about one third of patients with unrecognized MI also had ischemia [28]. In our study, the relatively high incidence of MACE and CVD mortality (11.4% and 6.8% during a median follow-up of 6 years, respectively) supports a strategy of accurate risk stratification for CAD in patients with CKD. Interestingly, although patients with ischemia in the current study had a slightly lower BMI value, they had a higher proportion of diabetes with a lower eGFR value and a greater time with CKD—which underlines the important risk of developing obstructive CAD in diabetic patients with severe long-standing CKD. In line with several reports indicating that the clinical presentation of CAD is often atypical in CKD patients [5], the ischemia group included a higher proportion of patients with dyspnea on exertion and lower prevalence of angina, which can also be explained by the higher rate of patients with unrecognized MI with possible early signs of HF.

Different imaging modalities have been used to diagnose obstructive CAD in patients with known CKD [4, 29]. Stress CMR appears to be one of the most effective and safe modalities [30]. Besides its operator-dependence and potential lack of echogenicity, stress echocardiography is often limited in CKD patients by submaximal exercise or poor tolerance to high dose dobutamine tests [31]. SPECT radionuclide perfusion imaging may be hampered by artefacts associated with left bundle branch block [31]. Therefore, stress echo and SPECT may have only relative accuracy for detecting CAD in CKD, with a higher rate of both false-negative and false-positive tests [31, 32]. Coronary computed tomographic angiography has some limitations in CKD patients including the presence of coronary calcifications causing ‘blooming artefacts’ and the risk of renal dysfunction related to the iodinated contrast dye load [4, 32].

### Risk stratification of patients with known CKD

Although the predicted risks of patients with CKD are well below their observed risk, CAD risk assessments (e.g., from the pooled cohort equation) are based on general population studies [4]. Therefore, CKD patients exemplify the short-comings of risk assessment from population data. Whereas standard clinical guidelines recognize CKD as a “modifying factor” when using the standard risk equations [33, 34], they do not formally incorporate kidney-specific variables, even though eGFR is readily available. Although risk classification can be improved by adding kidney-specific variables such as eGFR [35], these prediction models provide relatively poor reclassification yield in patients with CKD compared to the general population [4, 35, 36]. To address this limitation, several other markers have been assessed including coronary artery calcium (CAC) score. Coronary artery calcification can facilitate primary prevention decisions in the CKD population [33]. Although the progression of coronary calcification is faster with worsening CKD, the prognostic value of CAC score is likely similar to that in the general population without additional value in this specific population [37]. Consistently, the prognostic value of several biomarkers, such as C-reactive protein, cardiac troponin, and BNP, seem to be similar to that of the general population [38].

Although several studies have shown an incremental prognostic value of stress CMR above traditional CVD risk factors in patients with suspected or known CAD [7, 8], its additional prognostic value in CKD patients is not well established. The current data show that stress CMR has accurate prognostic value for predicting MACE and CVD mortality in CKD patients, with an excellent safety profile. In line with others [28], unrecognized MI was also independently associated with the occurrence of MACE in those patients and improved the prediction risk model of MACE over traditional risk factors including eGFR. Moreover, using propensity score-matching, the prognostic value of the presence of ischemia and unrecognized MI were more than twofold higher in CKD 3 patients compared to patients with eGFR ≥ 60 ml/min/1.73 m^2^ after adjustment for traditional risk factors. All these findings suggest a relevant clinical interest of stress CMR for improved risk stratification in this specific population and may have implications for improved secondary prevention of known CKD patients.

These data may have implications for improved primary and secondary prevention of CKD patients. Indeed, the incremental prognostic value of stress CMR could help improve prevention strategies. There are obviously significant opportunities to improve the detection and treatment of established risk factors in CKD patients, and the Kidney Disease Improving Global Outcomes (KDIGO) Work Group proposed clinical practice guideline for the diagnosis, evaluation, prevention, and treatment of Chronic Kidney Disease-Mineral and Bone Disorder [33], and for lipid management in CKD [34]. Although historically a risk of nephrogenic systemic fibrosis has been described with Gadolinium-based contrast agents in severe CKD patients, recent reports specifically indicate that for group II gadolinium-based contrast agents, the risk of nephrogenic systemic fibrosis is sufficiently low or non-existent that routine renal function screening is not necessary. Along with its added prognostic value, the steadily increasing expertise and availability of stress CMR makes it a safe, reproducible, and reliable test to stratify the risk of cardiovascular events in patients with known CKD.

### Study limitations

First, 8.7% patients were lost to follow-up, which can be explained by the relatively long follow-up and the design of the study. However, the French National Registry of Death has been carefully reviewed, which strengthens the data on mortality. Second, baseline and follow-up data for medications, in particular the prescription of sodium-glucose cotransporter-2 (SGLT2) inhibitors which dramatically decreases CKD progression, and renal replacement therapy were not collected in the study. Also, the potential consequences on outcomes of the changes in decision-making due to stress CMR could not be collected in this retrospective study. Although the recent definition of CKD also incorporates albuminuria, it was not collected systematically in the current study. Symptoms were assessed by the sole presence of symptomatic angina or dyspnea on exertion without standardized classification. Data on the recurrence of angina were not collected during follow-up. During the inclusion period, patients with eGFR < 30 ml/min/1.73 m^2^ were excluded due the risk of nephrogenic systemic fibrosis. However, the risk of nephrogenic systemic fibrosis is exceedingly low with current gadolinium-based contrast agents and the most recent American College of Radiology guidelines clearly state that eGFR < 30 does not necessarily preclude administration of gadolinium [39]. Although the use of two vasodilator agents (dipyridamole and adenosine) on each of the two recruiting centers may introduce protocol heterogeneity and an unbalanced recruitment between the two centers, the prognostic value of stress CMR remains homogeneous and constant in the two centers regardless of the vasodilator agent used. In addition, the current perfusion protocol includes a total of 6 views (4 short-axis, 2 chamber, and 4 chamber views) with an acquisition performed every two heartbeats with the aim of offering the best spatial LV coverage. However, this CMR protocol is different from that of the current guidelines [12], with data acquisition at each heartbeat with a risk of decreasing the temporal resolution. Of note, left atrial size and volume were not collected. Furthermore, the proportion of patients with unrecognized MI in the current study was slightly elevated, limiting the applicability of the current findings to other populations with CKD. Although it is important to mention that current guidelines also allow assessment of the presence of obstructive CAD using invasive coronary angiography in these symptomatic CKD patients with multiple risk factors, the use of stress CMR limits the risk of iodine contrast injection. Finally, as previously published by our working group [16, 17], the determination of the number of segments with inducible ischemia and LGE was visual without quantitative methods and without resting perfusion, but it represents the most widely used clinical method with optimal diagnostic accuracy.

## Conclusions

Stress perfusion CMR has a good discriminative and incremental long-term prognostic value in symptomatic stage 3 CKD patients without known CAD. These data support the role of stress CMR in patients with stage 3 CKD for stratifying the risk of CVD events. Whether those findings could result in advances in decision making and ultimately turn into clinical benefits needs further evaluation.

## Supplementary Information

**Additional file 1.** Supplemental files.

## Data Availability

All data generated or analysed during this study are included in this published article [and its Additional file 1: supplementary information files].

## Abbreviations

AF: Atrial fibrillation
bSSFP: Balanced steady state free precession
BNP: Brain natriuretic protein
CAD: Coronary artery disease
CKD: Chronic kidney disease
CMR: Cardiovascular magnetic resonance
CVD: Cardiovascular disease
ECG: Electrocardiogram
HF: Heart failure
HR: Hazard ratio
IQR: Interquartile range
LGE: Late gadolinium enhancement
LV: Left ventricle/left ventricular
LVEF: Left ventricular ejection fraction
MACE: Major adverse clinical events
MI: Myocardial infarction
NRI: Net reclassification index
SPECT: Single proton emission computed tomography
