# Supplementary material for: Safety and incremental prognostic value of stress cardiovascular magnetic resonance in patients with known chronic kidney disease
Source: J Cardiovasc Magn Reson. 2023 Jun 12;25:29. doi: 10.1186/s12968-023-00939-8 (PMC10259036; doi:10.1186/s12968-023-00939-8)
Supplement: Supplementary file 1 — Additional file 1. Supplemental files. [file 12968_2023_939_MOESM1_ESM.docx]

**Supplemental Files**

**Table of Contents:**

- **Supplemental file 1.** List of exclusion criteria.
- **Supplemental file 2.** Definition of clinical events.
- **Supplemental file 3.** Cardiovascular magnetic resonance (CMR) Protocol.
- **Supplemental file 4.** CMR sequence parameters.
- **Supplemental file 5.** Safety results.
- **Supplemental file 6.** Kaplan-Meier curves for MACE stratified by the presence of inducible ischemia for each recruitment center.
- **Supplemental file 7**. Univariable and multivariable analyses of inducible ischemia for prediction of adverse events (N=702).
- **Supplemental file 8.** Multivariable Cox regression analysis for the prediction of MACE in patients with eGFR between 30 and 60 ml/min/1.73 m^2^ using the propensity-matched population.

**SUPPLEMENTAL FILE 1**

**List of exclusion criteria**

Exclusion criteria were: (1) patients with a known stenosis ≥50% on at least 1 epicardial coronary artery on invasive coronary angiography or computed tomography angiography, patients with a positive functional test; (2) patients with a history of MI, defined by a history of MI on the medical records or presence of significant Q wave on 12-lead ECG in a coronary territory; (3) prior revascularization [percutaneous coronary intervention or coronary artery bypass graft]; (4) history of acute hospitalization for heart failure defined by the use of intravenous diuretics, or known LV dysfunction (defined by LVEF<50%); (5) known peripheral atheroma; (6) history of atrial fibrillation on the medical records or deﬁned by 12-lead electrocardiogram before and after CMR with consensus between 2 senior cardiologists; (7) any cardiovascular-related symptoms such as chest pain or shortness of breath at rest or on exertion 6 months prior to enrollment; (8) contraindication to CMR (cerebral clips, metallic eye implant); (9) contraindication to dipyridamole (severe asthma or chronic obstructive pulmonary disease, second- or third-degree atrioventricular block); (10) known cardiomyopathy (e.g. hypertrophic, dilated, or infiltrative) and acute or chronic myocarditis; (11) contraindications to CMR (cerebral clips, metallic eye implants); (12) known allergy to gadolinium-based contrast medium; and (13) glomerular filtration rate <30 mL/min/1.73 m^2^.

**SUPPLEMENTAL FILE 2**

**Definition of clinical events**

- Nonfatal MI: was defined by typical angina of ≥ 20 min duration, ECG changes, and a rise in troponin or creatine kinase level above the 99 percentile of the upper reference limit^1^.
- Cardiovascular mortality: was defined as sudden cardiac death with documented fatal arrhythmias or any death immediately preceded by acute MI, acute or exacerbation of heart failure, or stroke.
- All-cause mortality: was defined using the electronic French National Registry of Death (*Institut National de la Statistique et des Etudes Economiques*, INSEE registry).
- Late coronary revascularization was defined by a revascularization occurring > 90 days after CMR. For patients who underwent PCI within 90 days after the index examination, peri-procedural events (MI or cardiovascular mortality)^2^ were not included in the analysis.
- Hospitalization for heart failure: was defined by a previous history of hospitalization with symptoms and/or signs of HF with evidence of diastolic or systolic dysfunction by echocardiography and elevated levels of natriuretic peptide (BNP > 35 pg/ml and/or NT-proBNP > 125 pg/ml), according to guidelines^3^.
- Ventricular tachycardia was defined as documented sustained ventricular tachycardia on 12-leads ECG.
- Finally, all clinical events were defined according to standardized definitions^4^.

References

1. Thygesen K, Alpert JS, Jaffe AS, et al., Executive Group on behalf of the Joint European Society of Cardiology (ESC)/American College of Cardiology (ACC)/American Heart Association (AHA)/World Heart Federation (WHF) Task Force for the Universal Definition of Myocardial Infarction. Fourth Universal Definition of Myocardial Infarction (2018). Circulation. 2018;138:e618–e651.

2. Stone GW, Ben-Yehuda O, Sabik JF, et al. Considerations for an optimal definition of procedural myocardial infarction. Eur Heart J. 2020;41:1704–1705.

3. Ponikowski P., Voors AA., Anker SD., et al. 2016 ESC Guidelines for the diagnosis

and treatment of acute and chronic heart failure: The Task Force for the diagnosis and

treatment of acute and chronic heart failure of the European Society of Cardiology

(ESC) Developed with the special contribution of the Heart Failure Association (HFA) of the ESC. Eur Heart J 2016;37(27):2129–200. Doi: 10.1093/eurheartj/ehw128.

4. Hicks KA, Tcheng JE, Bozkurt B, et al. 2014 ACC/AHA Key Data Elements and Definitions for Cardiovascular Endpoint Events in Clinical Trials. Journal of the American College of Cardiology. 2015;66:403–469.

**SUPPLEMENTAL FILE 3**

**Cardiovascular magnetic resonance (CMR) Protocol**

CMR was performed using a 1.5T scanner (MAGNETOM Espree, and MAGNETOM Aera, Siemens Healthcare, Erlangen, Germany for Institut Cardiovasculaire Paris Sud; and MAGNETOM Avanto and Aera, Siemens Healthcare, Erlangen, Germany for Lariboisiere University Hospital). Long-axis (2-, 3-, and 4-chamber) and short-axis cine images encompassing the left ventricle from base to apex were obtained with a segmented retrospectively gated balanced steady state free precession (b-SSFP) sequence.^1^ In case of arrythmia, we used a segmented retrospectively gated balanced steady-state free precession sequence with an arrhythmia rejection algorithm. The mean heartbeat duration was determined over a period of 30 s. R waves were rejected for RR intervals lying outside a window (-25% to +25%) from the mean RR interval. If the pacemaker produced significant artifacts that could hamper image analysis, a gradient echo sequence (FGRE) was used.

Vasodilatation was induced with dipyridamole injected at 0.84 mg/kg over 3 min for all patients in Institut Cardiovasculaire Paris Sud, and with adenosine (Adenoscan, Astellas Pharma US, Deerfield, Illinois) infused at a rate of 140 mg/kg/min over 6 min for all patients in Lariboisiere University Hospital. At the end of vasodilator agent infusion, a bolus of gadolinium-based contrast agent (Dotarem, Guerbet, France, 0.1 mmol/kg) was injected at a rate of 5.0 ml/s with an injector (Mallinckrodt Optistar^®^ Elite). The stress perfusion images were acquired during 35 repetitions of the 6 slices, which allowed gadolinium first-pass imaging in all patients. Stress perfusion imaging was performed using an electrocardiogram (ECG)-triggered saturation-prepared balanced steady-state free-precession (b-SSFP) sequence with the following typical parameters: repetition time/echo time (TR/TE)=287/1.2 ms, acceleration factor=2, field of view=370x314 mm, matrix=224×180, reconstructed pixel size =1.7×1.7×8 mm. A series of six slices (four short-axis views, a 2-chamber, and a 4-chamber view) were acquired every other heartbeat. For quality check purposes, 2 repetitions of the 6 LV locations were acquired before gadolinium injection. In case of artifacts or wrong slice positioning, the parameters were adapted and a new test sequence was acquired until the quality check was validated. To limit through-plane motion, the patient was asked to have shallow breathing. Then, theophylline was injected intravenously to null the effect of dipyridamole. Ten minutes after contrast injection, breath-hold contrast-enhanced 3D T1-weighted inversion-recovery gradient-echo sequences were acquired to detect late gadolinium enhancement (LGE). The inversion time was individually adjusted to null normal myocardium. In case of arrythmia-induced artifacts on LGE, additional 2-dimensional single-shot with phase-sensitive inversion-recovery reconstructions were acquired. No rest perfusion imaging was performed in accordance with current guidelines.^1^ Patients were asked to refrain from caffeine at least 12 h before CMR. Safety was studied with clinical monitoring one hour after CMR. A 12-lead ECG was performed both before and after CMR examination.

References

1. Kramer CM, Barkhausen J, Bucciarelli-Ducci C, Flamm SD, Kim RJ, Nagel E. Standardized cardiovascular magnetic resonance imaging (CMR) protocols: 2020 update. J Cardiovasc Magn Reson. 2020;22:17.

**SUPPLEMENTAL FILE 4**

**Table. CMR sequence parameters.**

|  | **Cine**  **long-axis** | **Perfusion** | **Cine**  **short-axis** | **TI Scout** | **LGE** |
| --- | --- | --- | --- | --- | --- |
| **Pulse sequence** | b-SSFP | Saturation-recovery b-SSFP | b-SSFP | b-SSFP | 3D inversion-recovery fast gradient echo (FGRE) |
| **Field of view (FoV)** | 380 x 340 mm² | 370 x 314 mm² | 380 x 304 mm² | 380 x 307 mm² | 340 x 340 mm² |
| **Slice thickness** | 6 mm | 8 mm | 8 mm | 8 mm | 6 mm |
| **Matrix size** | 256 pixels | 224 pixels | 240 pixels | 208 pixels | 272 pixels |
| **Phase resolution** | 95 % | 80 % | 95 % | 100 % | 89 % |
| **Slice resolution** | NA | NA | NA | NA | 67 % |
| **Phase oversampling** | 0 % | 0 % | 0 % | 0 % | 20 % |
| **Slice oversampling** | NA | NA | NA | NA | 20 % |
| **Voxel size (reconstructed)** | 1.5 x 1.5 x 6 mm^3^ | 1.7 x 1.7 x 8 mm^3^ | 1.6 x 1.6 x 8 mm^3^ | 1.8 x 1.8 x 8 mm^3^ | 1.3 x 1.3 x 6 mm |
| **Voxel size (acquired)** | 1.5 x 1.6 x 6 mm^3^ | 1.7 x 2.0 x 8 mm^3^ | 1.6 x 1.7 x 8 mm^3^ | 1.8 x 1.8 x 8 mm^3^ | 1.3 x 1.4 x 8 mm |
| **TE/TR** | 1.18/2.9 ms | 1.04/2.8 ms | 1.15/2.8 ms | 1.31/3 ms | 1.35/3.4 ms |
| **Flip angle (FA)** | 55° | 70° | 55° | 30° | 9° |
| **Acceleration** | 6.1  (CS) | 2  (GRAPPA) | 6.5  (CS) | 4.2  (CS) | 2  (GRAPPA) |

Abbreviations: bSSFP: balanced steady-state free-precession; CS: compressed-sensing; FGRE: fast gradient echo; FoV: reconstructed field of view; LGE: late gadolinium enhancement; TE: echo time; TI: inversion time; TR: repetition time, NA: not applicable.

**SUPPLEMENTAL FILE 5**

**Safety results**

There were three cases of unstable angina, but no transient ischemic attack, disabling stroke, ST elevation MI or sustained ventricular tachycardia in relation to stress CMR.

Among the 769 patients who completed the stress CMR protocol, the main adverse events during or immediately after the study were as follows: 112 headaches (14.6%), 78 chest discomforts due to dipyridamole (10.1%), 72 nausea or vomiting (9.4%), 23 angina with ECG evidence of ischemia (3.0%), and 21 dizziness (2.7%). For all patients, symptoms resolved quickly with intravenous theophylline and additional sublingual nitrates and/or intravenous betablockers in 19 patients (2.5%).

**SUPPLEMENTAL FILE 6**

**Kaplan-Meier curves for MACE stratified by the presence of inducible ischemia for each recruitment center.**

Abbreviations: MACE: major adverse clinical events.


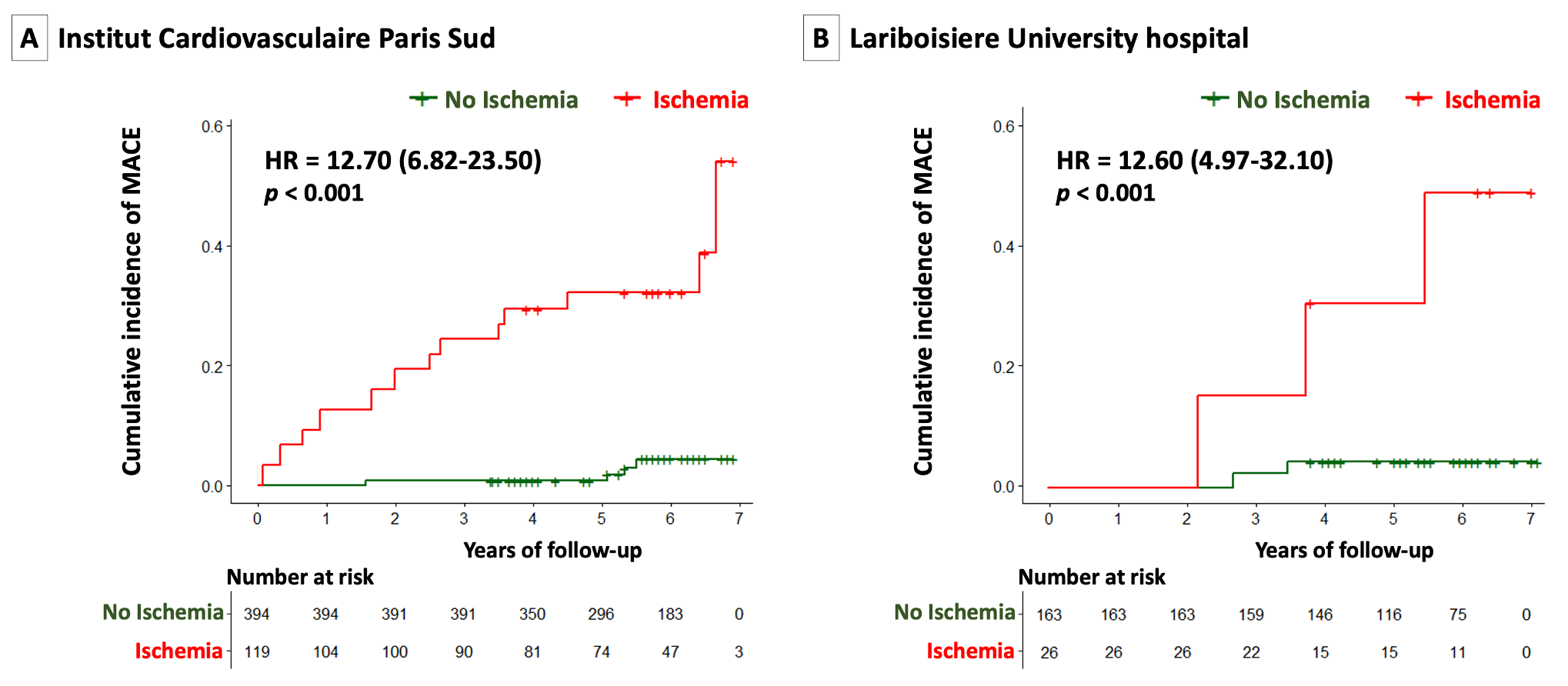


**SUPPLEMENTAL FILE 7**

**Table. Univariable and multivariable analyses of inducible ischemia for prediction of adverse events (N=702).**

|  | **Univariable analysis** | |  | **Multivariable analysis*** | |
| --- | --- | --- | --- | --- | --- |
|  | **Hazard Ratio**  **(95% CI)** | **p value** |  | **Hazard Ratio**  **(95% CI)** | **p value** |
|  |  |  |  |  |  |
| ***Primary outcome (MACE)*** |  |  |  |  |  |
| Cardiovascular mortality | 9.76 (5.19-18.4) | **<0.001** |  | 7.67 (2.61-22.6) | **<0.001** |
| Nonfatal MI | 16.9 (6.92-37.3) | **<0.001** |  | 8.33 (4.31-10.7) | **<0.001** |
|  |  |  |  |  |  |
| ***Secondary outcomes*** |  |  |  |  |  |
| All-cause of mortality | 2.56 (1.70-3.86) | **<0.001** |  | 1.51 (0.93-2.43) | 0.094 |
| Hospitalization for heart failure | 1.60 (0.97-2.65) | 0.065 |  | 1.14 (0.65-2.00) | 0.645 |
|  |  |  |  |  |  |

* Model included traditional CV risk factors: age, male, BMI, diabetes mellitus, hypertension, dyslipidemia, current or previous smoking, family history of CAD, LVEF per 10%, time between CKD diagnosis and CMR exam, unrecognized MI, and inducible ischemia.

*Abbreviations*: CI: confidence interval; HR: hazard ratio; LVEF: left ventricular ejection fraction; MACE: major adverse cardiac events; MI: myocardial infarction.

**SUPPLEMENTAL FILE 8**

**Multivariable Cox regression analysis for the prediction of MACE in patients with eGFR between 30 and 60 ml/min/1.73 m^2^ using the propensity-matched population.**

|  | **Patients**  **with**  **eGFR between 30 and 60 ml/min/1.73 m^2^**  **(N=702)** | |  | **Propensity-matched patients**  **with**  **eGFR ≥ 60 ml/min/1.73 m^2^**  **(N=702)** | |
| --- | --- | --- | --- | --- | --- |
|  | **Hazard Ratio**  **(95% CI)** | **p value** |  | **Hazard Ratio**  **(95% CI)** | **p value** |
| Model 1* |  |  |  |  |  |
| Age | 1.02 (0.99-1.06) | 0.17 |  | 1.05 (1.01-1.07) | **0.03** |
| Male | 0.86 (0.51-1.48) | 0.59 |  | 1.20 (0.93-1.89) | 0.06 |
| Body mass index | 1.05 (0.99-1.13) | 0.12 |  | 1.07 (0.95-1.22) | 0.08 |
| Diabetes mellitus | 5.22 (0.88-9.50) | **<0.001** |  | 2.50 (1.32-5.02) | **<0.001** |
| Hypertension | 3.66 (1.99-6.73) | **<0.001** |  | 1.9 (1.12-3.71) | **<0.001** |
| Dyslipidemia | 0.77 (0.51-1.25) | 0.63 |  | 1.00 (0.77-1.31) | 0.98 |
| Current or previous smoking | 2.81 (1.55-5.11) | **<0.001** |  | 0.97 (0.66-1.39) | 0.83 |
| Family history of CAD | 1.08 (0.47-2.46) | 0.78 |  | 1.29 (0.76-2.17) | 0.35 |
| LVEF | 0.92 (0.72-1.19) | 0.54 |  | 0.89 (0.77-0.97) | **0.03** |
| eGFR | 0.83 (0.61-0.97) | **0.03** |  | 0.78 (0.57-0.92) | **0.02** |
| Time between CKD diagnosis and CMR exam | 1.62 (0.91-2.59) | 0.19 |  | - | - |
|  |  |  |  |  |  |
| Model 2^†^ |  |  |  |  |  |
| Presence of unrecognized MI | 5.07 (3.13-8.21) | **<0.001** |  | 1.90 (1.30-2.62) | **<0.001** |
|  |  |  |  |  |  |
| Model 3^‡^ |  |  |  |  |  |
| Presence of unrecognized MI | 4.67 (2.83-7.68) | **<0.001** |  | 1.70 (1.22-2.51) | **0.003** |
| Presence of inducible ischemia | 15.5 (7.72-30.9) | **<0.001** |  | 3.74 (2.77-5.26) | **<0.001** |
|  |  |  |  |  |  |

* **Model 1** included traditional CV risk factors: age, male, BMI, diabetes mellitus, hypertension, dyslipidemia, current or previous smoking, family history of CAD, LVEF, eGFR and time between CKD diagnosis and CMR exam.

† **Model 2** included: model 1 with unrecognized MI.

‡ **Model 3** included: model 2 with inducible ischemia.

*Abbreviations: BMI: body mass index; CAD: coronary artery disease; CI: confidence interval CV: cardiovascular; LGE: late gadolinium enhancement; MACE: major*
